# Targeting COX-2 to Alleviate Depressive Phenotypes: Etoricoxib’s Antidepressant Effects in Chronic Unpredictable Mild Stress-Exposed Mice

**DOI:** 10.7759/cureus.99282

**Published:** 2025-12-15

**Authors:** Santhanalakshmi P, Manimekalai K, Sathiyamoorthy P

**Affiliations:** 1 Pharmacology, Mahatma Gandhi Medical College and Research Institute, Sri Balaji Vidyapeeth (Deemed to be University), Puducherry, IND; 2 Physiology, Pondicherry Institute of Medical Sciences, Puducherry, IND

**Keywords:** antidepressant, cox-2 inhibitors, cums, etoricoxib, sucrose preference test

## Abstract

The current study evaluated the antidepressant effect of etoricoxib, a well-established non-steroidal anti-inflammatory drug, in the chronic unpredictable mild stress (CUMS) model of depression in Swiss albino mice. The mice were exposed to CUMS for 42 days by using a variety of stressors. A total of 54 Swiss albino mice of either sex were used for the study, which were tested for locomotor activity using the Open Field Test (OFT). The mice were randomly divided into four groups, each with six animals, which were used to evaluate the antidepressant effect of etoricoxib using the Forced Swim Test (FST). For the prolonged experiment, the remaining 30 mice were used to evaluate the antidepressant effect using the chronic mild stress model (CMS), followed by a sucrose preference test (SPT) for six weeks. Treatment with etoricoxib at any of the doses (3 mg/kg and 9 mg/kg, PO) and the positive control, fluoxetine, significantly reversed these behavioral changes as compared to the CUMS using the OFT. The etoricoxib at 3 mg/kg and 9 mg/kg doses administered to stressed animals produced a significant (*p* < 0.05 and 𝑝 < 0.001) decrease in the duration of immobility when compared with CUMS control group. The test drug etoricoxib (3 mg/kg and 9 mg/kg) significantly (*p* < 0.001) increased the sucrose preference at week 6 when compared to the stress group. To conclude, the present study suggested that etoricoxib exerts an antidepressant-like effect in mice exposed to CUMS by reducing the symptoms of depression based on behavioural tests.

## Introduction

Major depressive disorder (MDD) and other mood disorders are psychiatric abnormalities characterized by the presence of emotional, cognitive, psychomotor, and neurovegetative symptoms. Due to its strong disability-related association and relatively high lifetime prevalence (2-15%), depression is a significant global public health concern [1]. Despite postulating so many hypotheses and mechanisms for depression, its exact pathogenesis still remains questionable. Several novel biological substrates for this illness have been postulated in addition to the known ones, such as the monoaminergic and serotonergic hypotheses.

The inflammatory and neurodegenerative hypothesis of depression was proposed as one potential explanation for these changes [2]. According to a recent concept, depression is characterized by the activation of the inflammatory response system, which results in increased production of proinflammatory cytokines such as interleukin (IL)-1b, IL-6, tumor necrosis factor-alpha (TNF-a), and prostaglandin E2 (PGE2) [3,4]. Moreover, therapy with proinflammatory agents also triggers depression-like symptoms [5]. So, studies have looked into whether anti-inflammatory medication use could enhance the effectiveness of antidepressants. Nonsteroidal anti-inflammatory medicines (NSAIDs), particularly the selective cyclooxygenase 2 (COX-2) inhibitor, celecoxib, exert anti-inflammatory effects by inhibiting pro-inflammatory cytokines. And also, NSAIDs inhibit COX-2, which also indirectly inhibits cytokine production [6].

Etoricoxib is an NSAID and analgesic known for its potent COX-2 inhibition. It is a COX-2 inhibitor (coxib) of the second generation loaded with so many advantages, such as high potency, more selective, rapid action, and reduction in peptic ulcer and gastrointestinal bleeding as compared to other coxibs. It is recommended for the short-term or long-term therapy of inflammation linked to musculoskeletal and joint conditions, such as osteoarthritis, rheumatoid arthritis, and so on [7].

Depression is produced chronically by a chronic mild stress (CMS) animal model of depression by inducing inflammatory and oxidative responses in rodents [8,9]. Chronic unpredictable stress has been linked to depressive-like behavior, and treatment with celecoxib has been shown to counteract this effect by lowering COX-2 expression in the brain and consequently the level of PGE2 [10]. Few studies used celecoxib as an additional therapy that has shown a significant reduction in depression symptoms. In all these studies, celecoxib, an anti-inflammatory drug, was proven to be effective as an antidepressant but only as an adjunctive drug along with fluoxetine, sertraline, and piroxicam, respectively [11-13].

To our knowledge, there is a lack of studies conducted using etoricoxib as an antidepressant and also coxib as a single therapeutic agent for depression. Therefore, taking into account the significant roles of COX-2 in the central nervous system and its probable participation in the pathophysiology of depression, the aim of the study is to evaluate the antidepressant effect of a potent, more selective, rapidly acting, and more tolerable coxib, etoricoxib, using Swiss albino mice.

## Materials and methods

Animals

Swiss albino mice (8-10 weeks of age) weighing 25-30 grams were procured from Biogen Laboratory Animal Facility, Bangalore, Karnataka, India, and housed in the Central Animal House of Mahatma Gandhi Medical College and Research Institute (MGMCRI), Puducherry, India. The study was performed after getting approval from the Institutional Animal Ethics Committee (IAEC) of MGMCRI (approval number: 03/IAEC/MG/01/2022-I). The study was conducted in compliance with the Committee for Control and Supervision of Experiments on Animals (CCSEA) guidelines.

The animals were quarantined for one week before the commencement of the study. The animals were maintained under standard laboratory conditions (light period of 12 hours/day and temperature of 23°C±2) with free access to food and water ad libitum. The animals were fed a standard chow diet and hygienic water. The study was performed once the animals were comfortable in the environment of the Central Animal House.

The mice were grouped as follows: Group 1 (n=6): Control (Distilled water); Group 2 (n=6): Treated with a standard antidepressant (fluoxetine 10 mg/kg PO); Group 3 (n=6): Treated with test drug - Dose 1 (Etoricoxib 3 mg/kg PO) [14]; Group 4 (n=6): Treated with test drug-Dose 2 (Etoricoxib 9 mg/kg PO) [14].

Experimental design

A total of 54 Swiss albino mice of either sex were used for the study. All the mice were tested for locomotor activity using the Open Field Test (OFT). Of the 54 mice, 24 were randomly divided into four groups with six animals in each, which were used to evaluate the anti-depressant effect of etoricoxib by the Forced Swim Test (FST) after an acute experiment, i.e., after one hour of the test and administration of the drugs. FST was performed only one day, and the changes in immobility duration were recorded. For the prolonged experiment, the remaining 30 mice were used to evaluate the antidepressant effect using the CMS model, followed by a sucrose preference test (SPT) for six weeks (42 days); for the initial 21 days, only CMS was given, followed by CMS along with standard and test drugs for the next 21 days [13,15]. 

Forced Swim Test

The test was conducted using a slightly modified method described by Porsolt et al. [16]. In a 5-liter glass cylinder that had 15 cm of water in it, each mouse was placed separately. Each mouse was permitted to swim for six minutes while being watched. Manual timekeeping was used to record the duration of immobility. The immobility time was calculated for the last four minutes of a six-minute test. When the mouse floats still or doesn't make any movements other than what is required to keep its head above the water's surface, it is regarded as immobile. Animals were taken out after the test and towel-dried. After each test, the water was replaced [13,17,18].

Chronic Unpredictable Mild Stress Model 

CMS was given to mice for six weeks. All animals were subjected to the mild stress protocol unpredictably for six weeks, without involving the mice in the no stress + control group. The protocol consisted of seven stressors, such as noise for three hours (high-pitch sounds), tilting the cage at 45° for seven hours, keeping it in light for eight hours during the night, crowded housing with eight mice occupying single cage for 12 hours, a soiled cage for 24 hours, food deprivation for 24 hours and water deprivation for 24 hours. All animals were housed singly, except for the crowded housing experiment. To prevent any habituation effect, the stressors were randomized every week [13,19].

The total duration of CUMS was six weeks (42 days). For the first 21 days, there was only CUMS for all mice except animals in the non-stressed group, using seven stressors, which were randomized every week. For the next 21 days, there was CUMS + drugs administered for the respective groups as mentioned earlier for all mice except animals in the non-stressed group. Results were analysed on the 42nd day. 

Sucrose Preference Test

The SPT was conducted every Monday morning for six weeks. The mice were kept single in a cage and administered 1%w/v solution of sucrose using two bottles. The following day, one bottle containing the 1% sucrose solution was replaced with a bottle containing drinking water for the next 24 hours for the animals to adapt to the sucrose solution. Sucrose preference tests were conducted by placing two pre-weighed bottles in each cage, one containing water and the other one containing 1%w/v sucrose solution. The animals were given a free choice to drink from either bottle. The animals were allowed to drink for one hour. The weights of both bottles were recorded, and the difference in their respective initial and final weights was calculated [13]. The percentage of sucrose preference was calculated based on the following formula: % Sucrose preference = Sucrose consumption × 100/Sucrose + Water consumption [19].

Open Field Test

The spontaneous locomotor activity of mice was evaluated by the following method. The apparatus consisted of a square box (75 cm × 75 cm with 42 cm height) made up of wooden material. The top of the box was not covered and kept open to observe the movement of the animal. The floor and all sides of the box were covered with cardboard material. The cardboard on the floor of the box was drawn with black lines dividing the floor into equal squares of 15 cm × 15 cm. The animals were placed in the centre of the box and allowed to explore the box freely for five minutes. The total number of squares crossed by the animals and the number of rearing were recorded [13,19].

Statistical analysis

All the parameters recorded from the above models were tabulated and expressed as mean ± standard error of the mean. One-way analysis of variance followed by post hoc Bonferroni correction was used for the analysis of data between different groups. For all inferential statistical tests, a P < 0.05 was considered to be statistically significant, and P < 0.01 was considered to be extremely statistically significant. GraphPad InStat software version 3.06 (Dotmatics, Boston, Massachusetts, United States) was used for the analysis of data.

## Results

Effect of etoricoxib and fluoxetine on the locomotor activity of mice in the OFT

**Figure 1:**
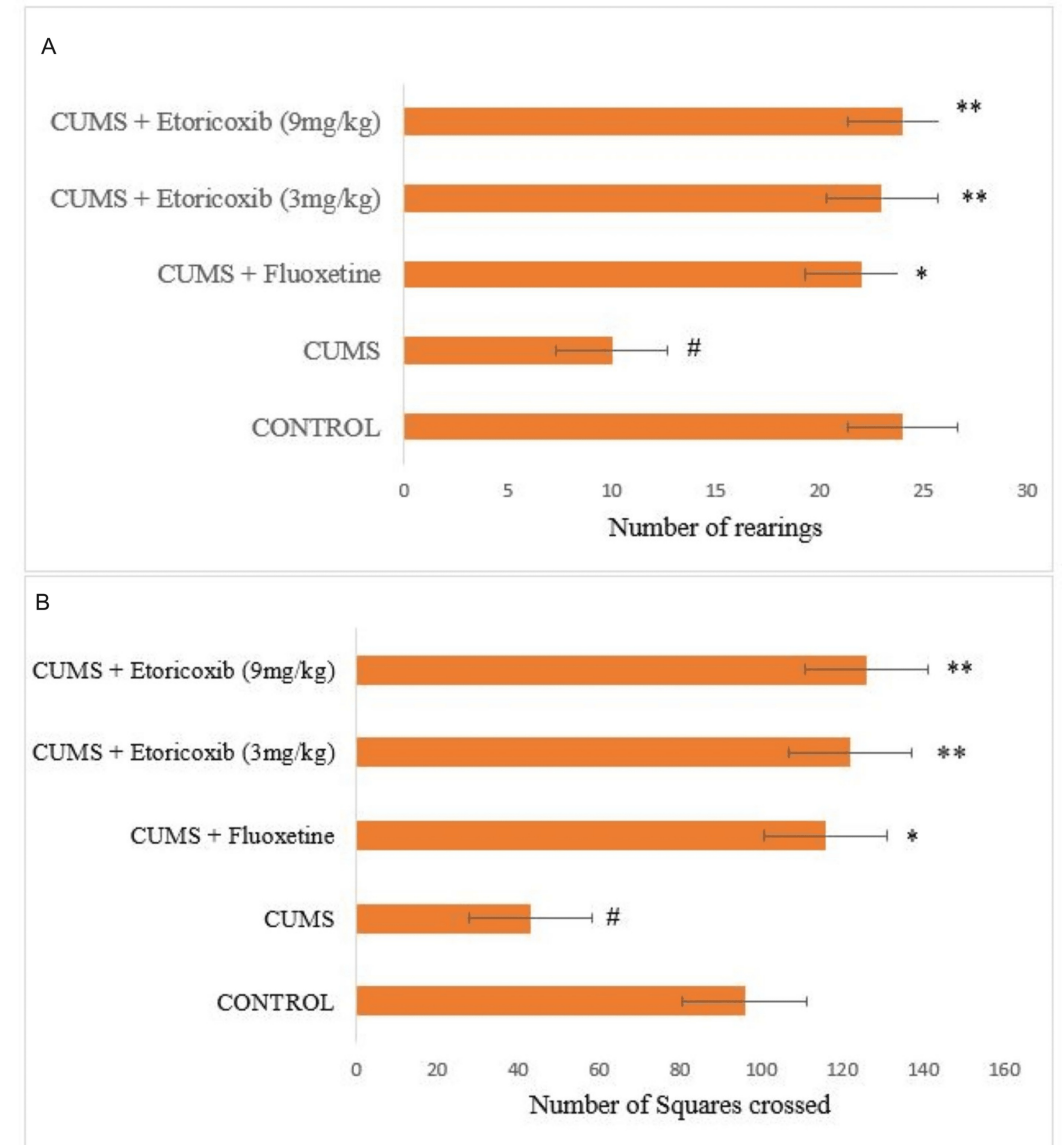
Effects of etoricoxib and fluoxetine on mice locomotion in the Open Field Test The error bars represent the standard errors of the means. ^#^p < 0.05 when compared with no stress group; ^∗^p < 0.05, ^∗∗^p < 0.01, and ^∗∗∗^p < 0.001 when compared with CUMS control group; one-way ANOVA followed by post hoc Bonferroni correction. CUMS: chronic unpredictable mild stress

Figures 1A, 1B show that the mice in CUMS group showed decreased activity in (A) the number of rearings and (B) the number of squares crossed, respectively, when compared to the control group. Treatment with etoricoxib at any of the doses (3 mg/kg and 9 mg/kg, PO) and the positive control, fluoxetine, significantly reversed these behavioral changes as compared to the CUMS using the OFT. 

Effect of etoricoxib and fluoxetine on the immobility time of mice in FST

**Figure 2:**
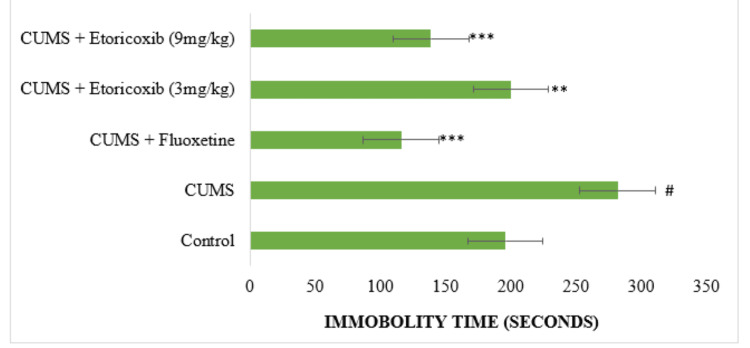
Effect of etoricoxib and fluoxetine on mice immobility duration in Forced Swim Test Data represent mean ± SEM (𝑛=6). ^#^p < 0.05 when compared with no stress group; ^∗^p < 0.05, ^∗∗^p < 0.01, and ^∗∗∗^p < 0.001 when compared with CUMS control group; one-way ANOVA followed by post hoc Bonferroni correction test. SEM: standard error of the mean; CUMS: chronic unpredictable mild stress

Significant differences between the treated groups were noted with a p-value < 0.001. Post hoc analysis indicated that the CUMS control group showed an increase in the immobility time compared with the no-stress group (p<0.01). The etoricoxib at 3 mg/kg and 9 mg/kg doses administered to stressed animals produced a significant (p < 0.05 and p < 0.001) decrease in the duration of immobility when compared with CUMS control group (Figure 2). Fluoxetine also significantly reduced the duration of immobility (p < 0.001) when compared to CUMS control group. 

Effects of CUMS and treatment with fluoxetine (10 mg/kg) or etoricoxib (3 mg/kg or 9 mg/kg) on SPT

**Figure 3:**
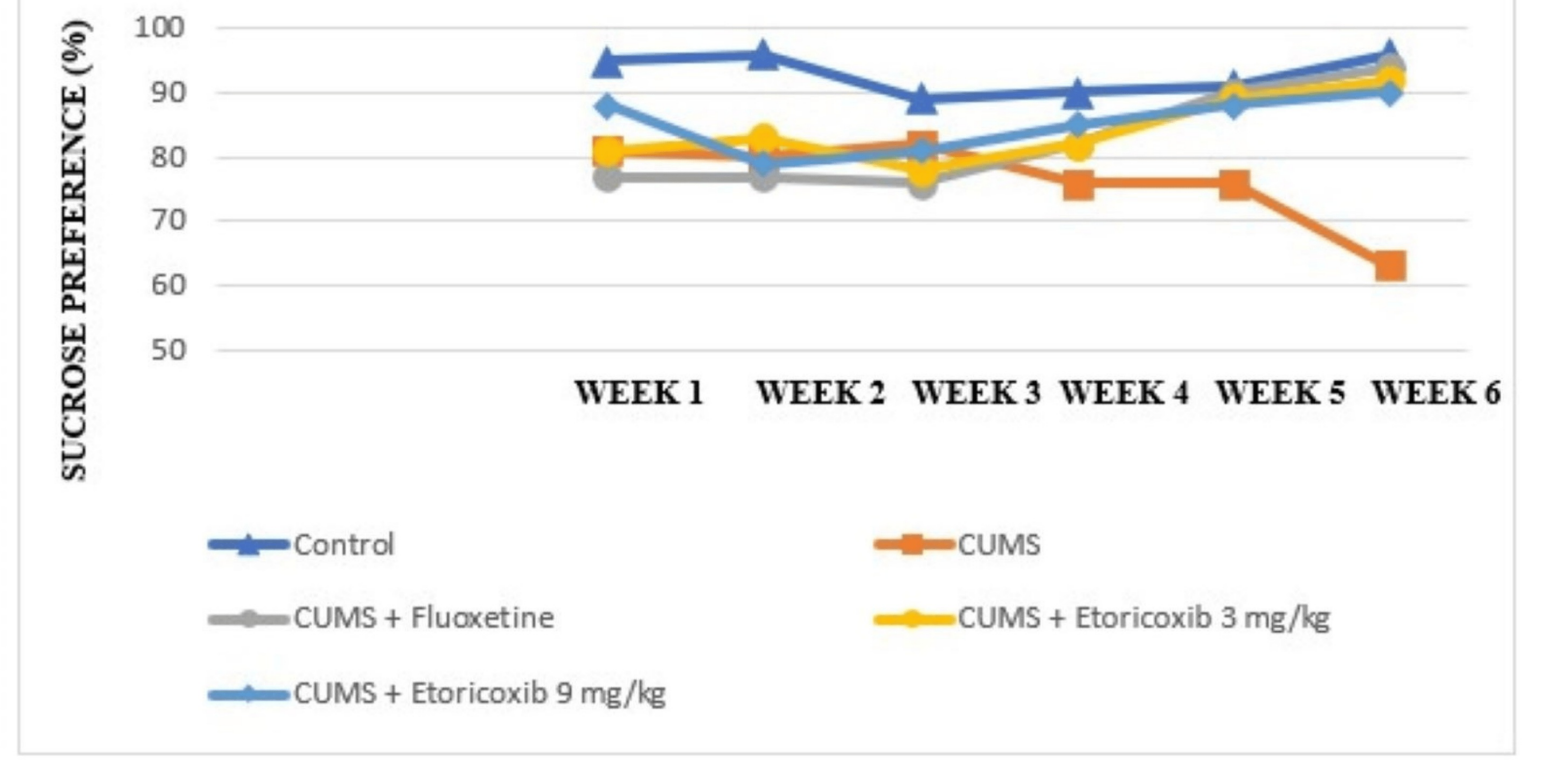
Effects of CUMS and treatment with fluoxetine and etoricoxib on the sucrose preference test

At the end of week 2, all mice that were subjected to CUMS protocol revealed a significant decline in sucrose preference when compared to the control group without stress (p < 0.001). The CUMS group show significant decrease in sucrose preference until week 6 (p < 0.01). The test drug etoricoxib (3 mg/kg and 9 mg/kg) significantly increased the sucrose preference at week 6 when compared to the stress group (p < 0.001) (Figure 3). The fluoxetine treatment, which began in week 3, steadily reversed the sucrose preference near their baseline levels by significantly (p< 0.05) increasing the sucrose preference at week 5 and 6 when compared to CUMS group. 

## Discussion

The mice were subjected to CMS for six weeks. CMS leads to specific behavioral abnormalities that resemble symptoms seen in human depression, and various antidepressants can reverse these symptoms. CMS-induced mice can effectively mimic the depressive state, as evidenced by reduced sucrose intake in SPT, decreased locomotor activity, and increased immobility time in FST, along with a reduction of neurotransmitters in the brain [20,21].

In the CMS model, mice were exposed to various stressors over a six-week period, including repeated mild physical and psychological stressors. After 21 days, the mice received treatment for CMS, which resulted in an increase in immobility time in the FST and a decrease in sucrose intake in the SPT. The behavioral change seen during the CMS procedure was replicated as in previous studies [22,23]. Our study also yielded parallel results with published studies, which showed that CMS exposure increased immobility time and decreased the mice's consumption of sucrose [21,23].

The FST was conducted after each week of the CMS procedure and at the end of the treatment period. Behavioral despair in mice was indicated by the considerable increase in immobility duration caused by stress during 21 days. In an animal model of depression, etoricoxib reduced the duration of immobility in both doses, with a more pronounced effect at the higher dose of 9 mg/kg compared to the lower dose (3 mg/kg). Similar results were also observed with other published articles using FST as an animal model of depression [24,25].

Additionally, anhedonia was identified using the SPT, which has been considered an index of anhedonia-like behavioral change in many studies. One of the main signs of depression is anhedonia brought on by CMS. Anhedonia is the inability to feel pleasure from activities that are often enjoyable. Compared to the normal group, stress dramatically decreased the preference for sucrose. Etoricoxib exhibited an increased preference for sucrose in both doses (3 mg/kg and 9 mg/kg), almost equal to the response given by the standard drug, fluoxetine, compared to the group treated by CUMS, suggesting the antidepressant like activity of etoricoxib [26,27]

To rule out any psychomotor stimulant activity, the locomotor activity of rodents was measured using the OFT. Compared to the control group, mice in the CUMS group exhibited reduced activity in both crossing and rearing behaviour. In our study, treatment with Etoricoxib at any of the doses (3 mg/kg and 9 mg/kg, PO) and the positive control, fluoxetine, significantly reversed these behavioral changes to normal when compared to the CUMS group. Psychomotor stimulants, convulsants, and anticholinergics are among the substances that tend to provide a false positive result because they increase locomotor activity in open field tests [28,29]. Antidepressants would not significantly increase motor activity, which is the main distinction between them and psychomotor stimulants. Hence, reversing the behavioural activities in the present study confirms the antidepressant-like activity of etoricoxib [30].

Interestingly, the results of our study clearly indicate that etoricoxib demonstrated antidepressant-like activity at both higher and lower test doses used in this research, with the most pronounced effect observed in the higher dose group of 9 mg/kg. Because of this antidepressant effect, patients receiving etoricoxib (which is an NSAID) for chronic ailments such as arthritis over the long term can better cope with the depression associated with pain from the disease or the prolonged therapy [31]. Due to its antidepressant effects and anti-inflammatory properties, excessive use of multiple drugs for various health-related ailments can be substituted with monotherapy, achieving similar outcomes.

## Conclusions

The present study suggested that etoricoxib exerts an antidepressant-like effect in mice exposed to CUMS by reducing the symptoms of depression based on behavioural tests. Further efforts are still required to validate the mechanisms behind the antidepressant effects of etoricoxib. Additionally, studies investigating its antidepressant effect in clinical subjects are warranted, particularly in patients with chronic disabling diseases or painful conditions such as arthritis or in those with depression.
